# A Novel Monocyte Subset as a Unique Signature of Atherosclerotic Plaque Rupture

**DOI:** 10.3389/fcell.2021.753223

**Published:** 2021-10-12

**Authors:** Ramona Vinci, Daniela Pedicino, Alice Bonanni, Alessia D’Aiello, Anna Severino, Eugenia Pisano, Myriana Ponzo, Francesco Canonico, Pellegrino Ciampi, Giulio Russo, Marianna Di Sario, Rocco Antonio Montone, Carlo Trani, Cristina Conte, Maria Chiara Grimaldi, Francesco Cribari, Massimo Massetti, Filippo Crea, Giovanna Liuzzo

**Affiliations:** ^1^Department of Cardiovascular and Pneumological Sciences, Catholic University of the Sacred Heart, Rome, Italy; ^2^Department of Cardiovascular Sciences, Fondazione Policlinico Universitario A. Gemelli IRCCS, Rome, Italy

**Keywords:** monocyte subsets, acute coronary syndromes, plaque rupture, inflammation, innate immunity

## Abstract

The evaluation of monocyte subset distribution among acute coronary syndrome (ACS) patients according to culprit coronary plaque morphology has never been explored. We evaluated whether there were significant differences in frequency of circulating monocyte subsets isolated from ACS patients according to optical coherence tomography (OCT) investigation of plaque erosion and rupture. We enrolled 74 patients with non-ST-elevation ACS (NSTE-ACS), 21 of them underwent OCT investigation of the culprit coronary plaque and local macrophage infiltration (MØI) assessment. As control, we enrolled 30 chronic coronary syndrome (CCS) patients. We assessed the frequency of monocyte subsets in the whole study population, in reliance on their CD14 and CD16 expression (classical, CM: CD14^++^CD16^–^; intermediates, IM: CD14^++^CD16^+^; non-classical, NCM: CD14^+^CD16^++^). Then, we tested the effect of lipopolysaccharide (LPS) (a CD14 ligand) on peripheral blood mononuclear cells (PBMCs) of NSTE-ACS patients, quantifying the inflammatory cytokine levels in cell-culture supernatants. Our data proved that monocyte subsets isolated from NSTE-ACS patients represent a peculiar biological signature of the pathophysiological mechanism lying beneath atherosclerotic plaque with a ruptured fibrous cap (RFC) as compared with plaque erosion. Moreover, the magnitude of LPS-mediated effects on IL-1β, IL-6, and IL-10 cytokine release in cell-culture supernatants appeared to be greater in NSTE-ACS patients with RFC. Finally, we described a fourth monocyte population never explored before in this clinical setting (pre-classical monocytes, PCM: CD14^+^CD16^–^) that was prevalent in NSTE-ACS patients as compared with CCS and, especially, in patients with RFC and culprit plaque with MØI.

## Introduction

Innate immunity activation represents a key mechanism in the pathogenesis of acute coronary syndromes (ACS), involved in the onset and progression of the atherosclerotic plaques (Shalhoub et al., 2011). Among the innate immunity cells, monocytes are the most plastic and dynamic ones, being able to switch toward several functional phenotypes (Canè et al., 2019). About two-thirds of ACS patients presenting with plaque rupture at optical coherence tomography (OCT) investigation had evidence of plaque macrophage infiltration (MØI) and raised systemic levels of inflammatory biomarkers (Scalone et al., 2017). In ACS patients with ruptured fibrous cap (RFC), monocyte-derived macrophages display thrombotic and inflammatory activity (Fracassi et al., 2021). Moreover, besides the monocyte-to-macrophage transition (Tabas and Lichtman, 2017), relying on CD14 (LPS co-receptor) and CD16 (Fc receptor FcRIII) surface expressions, three major monocyte subsets have been typically recognized, i.e., classical (CM: CD14^++^CD16^–^), intermediate (IM: CD14^++^CD16^+^), and non-classical (NCM: CD14^+^CD16^++^) (Passlick et al., 1989; Ziegler-Heitbrock et al., 2010), each one exhibiting a different pathophysiological role (Tsujioka et al., 2009; Ghattas et al., 2013; Mossanen et al., 2020; Oh et al., 2021). Our purpose was to evaluate monocyte subset distribution in NSTE-ACS patients according to OCT identification of plaque rupture and erosion.

## Materials and Methods

### Population, Sample Collection, and Cell Isolation

**FIGURE 1:**
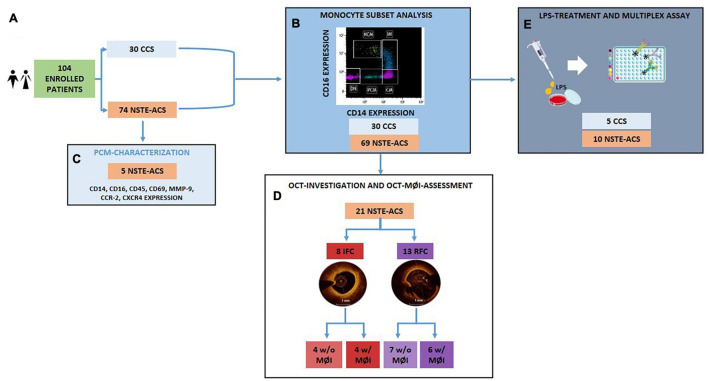
Flowchart showing the allocation of the enrolled patient population within each experimental procedure. **(A)** Among the 104 enrolled patients, 30 were CCS and 74 were NSTE-ACS; **(B)** The analysis of distribution of circulating monocyte subsets was performed according to CD14 and CD16 expression on 30 CCS and 69 NSTE-ACS patients; **(C)** 5 new NSTE-ACS patients were enrolled to characterize a novel monocyte subpopulation; **(D)** 21 of the 69 NSTE-ACS patients underwent OCT investigation and OCT-MØI assessment, resulting in 8 IFC and 13 RFC, respectively; **(E)** Lastly, 5 CCS and 10 NSTE-ACS patients of the 30 CCS and 69 NSTE-ACS, respectively, were employed for the *in vitro* LPS-treatment and cytokine multiplex assay. CCS, chronic coronary syndrome, IFC, intact fibrous cap; LPS, lipopolysaccharides; MØI, machrophage infiltration; NSTE-ACS, non ST-elevation acute coronary syndrome; PBMCs, peripheral blood mononuclear cells; RFC, ruptured fibrous cap; w/o, without; w/with. Art images from http://smart.servier.com.

Our population included 30 chronic coronary syndrome (CCS) patients and 74 patients with non-ST-elevation acute coronary syndromes (NSTE-ACS) (Knuuti et al., 2020; Collet et al., 2021). Characteristics of the study population are presented in Table 1, while patient allocation in each experimental condition is schematized in Figure 1. All patients gave their written informed consent. The local Ethics Committee approved the study. Exclusion criteria were patient age over 80 years; heart failure with reduced ejection fraction (HFrEF) [left ventricular ejection fraction (EF) <40%; New York Heart Association (NYHA) class III–IV] (Ponikowski et al., 2016); moderate to severe heart valve disease, both stenosis and regurgitation (inferred according to the ESC Guidelines, Baumgartner et al., 2017); in-stent restenosis cases or stent thrombosis; culprit saphenous vein graft lesions and more generally by-pass graft lesions (including left internal mammary artery, LIMA) due to the different pathogenic mechanisms with respect to native coronary lesions; anti-inflammatory or immunosuppressive therapies other than low-dose aspirin; autoimmune diseases; evidence of immunologic disorders or chronic infectious diseases; liver diseases (class A–C of CHILD-PUGH), malignancies; chronic kidney disease at stage 4; and recent major surgical procedures or trauma.

**TABLE 1:** Baseline characteristics of the study population.

	**CCS *n* = 30**	**NSTE-ACS *n* = 74**	***P* value**
Age, mean ± SD	69 ± 8.7	65 ± 12.9	0.131
Gender, M/F	22/8	54/20	0.970
**CV risk factors**			
Smoking (%)	19 (63)	46 (62)	0.911
Diabetes (%)	15 (50)	23 (33)	0.069
Hypertension (%)	26 (87)	55 (74)	0.168
Dyslipidemia (%)	16 (53)	38 (51)	0.855
Obesity (%)	7 (23)	12 (16)	0.395
Family history (%)	9 (30)	25 (34)	0.782
**Previous history**			
ACS (%)	8 (27)	28 (38)	0.359
Previous PCI (%)	8 (27)	23 (31)	0.299
Previous CABG (%)	0 (0)	5 (7)	0.320
**In-hospital management**			
LVEF ≥ 50% (%)	23 (77)	55 (74)	0.999
Multivessel disease (%)	7 (23)	29 (39)	0.171
PCI for the index event (%) CABG for the index event (%)	20 (77) 5 (17)	58 (7) 8 (11)	0.220 0.510
**Medical therapy**			
DAPT (%)^#^	10 (33)	29 (39)	0.944
ASA (%)	23 (77)	44 (60)	0.027
Clopidogrel (%)	10 (33)	16 (22)	0.146
Prasugrel (%)	0	1 (1)	0.535
Ticagrelor (%)	0	15 (20)	0.009
Anticoagulants (%)	0	5 (7)	0.157
Beta-Blockers (%)	20 (67)	41 (55)	0.301
Diuretics (%)	5 (17)	13 (17)	0.981
ACE-I (%)	15 (50)	30 (40)	0.375
ARBs (%)	3 (10)	20 (27)	0.075
Statins (%)	21 (70)	42 (57)	0.086
Calcium-channel blockers (%)	6 (20)	8 (11)	0.166
Nitrates (%)	0	1 (1)	0.535
Insulin (%)	5 (17)	7 (9)	0.232
Oral antidiabetic (%)	8 (27)	15 (20)	0.356
**Laboratory assay (mean ± SD)**			
Total cholesterol (mg/dL)	157 ± 41	158 ± 40	0.870
LDL (mg/dL)	91 ± 33	97 ± 34	0.620
HDL (mg/dL)	41 ± 9	41 ± 14	0.910
Triglycerides (mg/dL)	125 ± 44	130 ± 42	0.690
Monocyte count (10^9^/L)	0.5 ± 0.2	0.6 ± 0.2	0.146
Monocyte count (%)	6.5 ± 2.2	6.3 ± 1.7	0.685
hs-CRP (mg/L) (median and IRQ)	2.9 ± 12.6	10.5 ± 23.3	0.039
**Follow-up events**			
Recurrence of acute coronary events (%)	6 (20)	33 (45)	0.019
Cardiovascular death	0	2 (3)	–
Non-fatal MI	2 (7)	14 (19)	–
Ischemia-driven revascularization	4 (13)	17 (23)	–

*^#^These data refer to the time of patient enrollment and blood withdrawal. At the time of coronary angiography all the NSTE-ACS patients were on DAPT according to current guidelines.*

*Recurrence of acute coronary events means occurrence of cardiovascular death, non-fatal myocardial infarction, and ischemia-driven revascularization at 6–24 months of follow-up. Follow-up visits, consisting of physical examination, a standard 12-lead electrocardiogram, and a treadmill stress test were performed every 6 months.*

*CCS, chronic coronary syndromes; NSTE-ACS, non-ST-elevation acute coronary syndromes; SD, standard deviation; M/F, male/female; CV, cardiovascular; PCI, percutaneous coronary intervention; CABG, coronary artery bypass grafting; LVEF, left ventricular ejection fraction; DAPT, dual antiplatelet therapy; ASA, aspirin; ACE-I, Angiotensin-converting enzyme inhibitors; ARBs, angiotensin II receptor blockers; LDL, low-density lipoprotein; HDL, high-density lipoprotein; hs-CRP, high sensitive C-reactive protein; IQR, interquartile range; MI, myocardial infarction.*

Eighteen milliliters of venous blood were obtained at 9±3h from hospital admission and collected in ethylenediaminetetraacetic acid (EDTA) tubes. Peripheral blood mononuclear cells (PBMCs) were isolated at 2,200 × *g* and 25°C for 25 min through density gradient centrifugation methods (Lympholyte^®^-H Cell Separation Media, CEDARLANE). PBMC pellets were washed and suspended in Dulbecco’s phosphate-buffered saline (DPBS) (GIBCO; Invitrogen, Carlsbad, CA, United States) and used according to scheduled protocol steps (see sections “Flow Cytometry Analysis” and “Cell Cultures and Multiplex Assay Analysis”).

### Optical Coherence Tomography Investigation and Optical Coherence Tomography-Macrophage Infiltration Assessment

Optical Coherence Tomography images were obtained using a frequency domain OCT system (C7-XR FD-OCT; St. Jude Medical, St. Paul, MN, United States). OCT image analysis was performed using an offline review workstation (Ilumien Optis; St Jude Medical) by two expert investigators who were blinded to patient data. In presence of any diagnostic discordance between the two readers, a consensus was achieved taking into account the opinion of a third investigator. Plaque erosion has been classified by the presence of intra-luminal thrombus overlying a plaque presenting with intact fibrous cap (IFC), otherwise the presence of an irregular luminal surface at the site of the culprit lesion in the absence of thrombus. RFC has been defined by the existence of fibrous cap discontinuity with a cavity formation inside the plaque, or with a direct communication between the lumen and inner core of the lesion (Prati et al., 2010; Tearney et al., 2012; Johnson et al., 2019). Furthermore, the presence of macrophage infiltrations (MØI) at the culprit sites was assessed by OCT imaging. OCT-MØI appeared as “signal-rich, distinct, or confluent punctate regions that exceed the intensity of background speckle noise and generate a backward shadowing” (Phipps et al., 2015; Montone et al., 2020).

Among our NSTE-ACS patients who underwent OCT investigation of culprit coronary lesions before stent procedure for clinical reasons and in whom it was possible to clearly identify the features of the culprit plaques, eight presenting with IFC and 13 with RFC have been identified. Patients were then divided into four groups according to the presence or absence of MØI as follows: four IFC without MØI, four IFC with MØI, seven RFC without MØI, and six RFC with MØI.

### Flow Cytometry Analysis

The frequency of circulating monocyte subsets was assessed on basal PBMCs (30 CCS and 69 NSTE-ACS, of which 8 IFC and 13 RFC) by flow cytometry (Cytomics FC-500; Beckman Coulter, Brea, CA, United States). Monocyte staining with fluorochrome-labeled monoclonal antibodies (mAbs) was performed for 15 min at room temperature (RT) and under dark condition using anti-CD14 electron coupled dye (ECD)-conjugated and anti-CD16 phycoerythrin cyanin 7 (PC7)-conjugated (both from Beckman Coulter). Monocytes were gated using a forward/sideward scatter (FS/SS) plot, and monocyte subsets were defined according to their cluster of differentiations (CD) CD14 and CD16 surface expression (Figure 1A). Furthermore, to distinguish the monocyte populations, additional cell surface expressions of CD14, CD16, CD45, CD69, matrix metalloproteinase 9 (MMP-9), C-C chemokine receptor 2 (CCR2), and C-X-C motif chemokine receptor 4 (CXCR4) were simultaneously evaluated in five patients presenting with cardiovascular diseases using a multi-color detector configuration set up (CytoFlex S; Beckman Coulter). The relative median fluorescence intensity (MFI) was assessed, and monocyte subset distribution was shown as frequency (%) of each subset among the monocyte group according to gating strategy. Non-specific staining with isotype-matched control mAb was <1%. For each acquisition, 50,000 events were captured, and they were adequate for rare population analysis. Acquired data were analyzed using the Kaluza analysis software (Beckman Coulter).

### Cell Cultures and Multiplex Assay Analysis

PBMCs were incubated at a density of 2 × 10^6^/ml for 16 h at 37°C under 5% CO_2_ and 20% O_2_ in cell culture medium (RPMI 1640, LONZA) supplemented with 100 U/ml penicillin, 0.1 mg/ml streptomycin, 2 mmol glutamine and 10% fetal bovine serum (FBS; Thermo Fisher Scientific, Carlsbad, CA, United States) and treated (or not) with 1 μg/ml *Escherichia coli* lipopolysaccharide (LPS, Sigma-Aldrich, S. Louis, MO, United States), as CD14 ligand as well as a known pro-inflammatory stimulus. We then analyzed the concentrations of six cytokines (interleukin-1β, IL-1β; interleukin-1rα, IL-1rα; interleukin-6, IL-6; interleukin-8, IL-8; interleukin-10, IL-10; interferon-γ, IFN-γ) in collected supernatants from non-treated (NT) and LPS-treated PBMCs (*n* = 5 CCS and *n* = 10 NSTE-ACS, of which *n* = 4 IFC and *n* = 6 RFC) by using Bio-Plex^*TM*^ Pro human 6-plex for group I cytokine (Bio-Rad Laboratories, Hercules, CA, United States) according to the protocol provided by the manufacturer. Data were collected and analyzed using a Bio-Rad BioPlex 200 instrument equipped with Bio-Plex Manager software version 6.0 (Bio-Rad Laboratories, Hercules, CA, United States). The precision based on both intra- and inter-assay variations were <10% within the detection limits provided by the manufacturer.

### Statistical Analysis

Due to the pioneering nature of the study, it was not possible to make a formal estimate of the power and sample size. Therefore, the enrollment of 30 patients in the CCS group and 74 patients in the NSTE-ACS group was arbitrary. However, based on very recent data (Merah-Mourah et al., 2020), the differences we observed should maintain statistical significance even with increasing sample size.

Variable distribution was assessed by Shapiro–Wilk test. For data normally distributed, we used unpaired *t*-test for statistics between two groups and one-way ANOVA for repeated measures followed by Holm–Sidak’s multiple comparison test. Data that did not follow a normal distribution were analyzed using non-parametric tests, as follows: Mann–Whitney *U* test was performed for statistics between two groups, and Wilcoxon and Friedman or Kruskal–Wallis test followed by Dunn’s *post hoc* test for multiple comparisons. Mean ± standard deviation (SD) and median and interquartile range (IQR) have been used for description of normally and non-normally distributed data, respectively. For all the experimental assays, a two-tailed *p* ≤ 0.05 was considered statistically significant. The PRISM software (GraphPad 8.02; GraphPad Software Inc., San Diego, CA, United States) was used as statistics tool.

## Results

### Identification of a Novel Monocyte Subset

**FIGURE 2:**
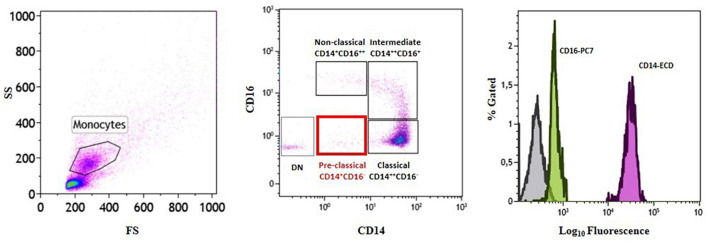
Identification of a novel subset of circulating monocytes.Gating strategy and monocyte subset identification by flow cytometry in a representative patient. Monocytes were gated in a forward/sideward scatter (FS/SS) dot plot (left plot) and the three monocyte subsets (classical, intermediate, and non-classical) (middle plot) as well as the novel *pre-classical* monocyte population were identified according to CD14 and CD16 cell surface expression (right histogram). DN = double negative.

Analysis of the distribution of circulating monocyte subsets according to their CD14 and CD16 surface expression led to the identification of a fourth monocyte population other than the three already known subsets (classical, CM: CD14^++^CD16^–^; intermediate, IM: CD14^++^CD16^+^; non-classical, NCM: CD14^+^CD16^++^). In line with CD14 and CD16 surface staining, the newly identified cells were CD14^+^CD16^–^ (Figure 2 and Table 2). We decided to label this novel subset as “*pre-classical monocytes*” (PCM) because of their proximity to the CM while observing the flow-cytometry acquired event distribution on dot plots.

**TABLE 2:** Monocyte phenotypic profiles.

**Monocyte subset**	**Phenotype**
Pre-classical monocyte	CD14^+^CD16^–^CD45^+^CD69^+^MMP9^+^CCR2^+^CXCR4^+^
Classical monocyte	CD14^++^CD16^–^ CD45^+^CD69^+^MMP9^+^CCR2^+^CXCR4^+^
Intermediate monocyte	CD14^++^CD16^+^CD45^++^CD69^+^MMP9^+^CCR2^+^CXCR4^+^
Non-classical monocyte	CD14^+^CD16^++^CD45^++^CD69^+^MMP9^+^CCR2^+^CXCR4^+^

*A more accurate flow-cytometry characterization of monocyte subsets was performed in five patients presenting with NSTE-ACS, according to the expression of the following surface markers: CD14, CD16, CD45, CD69, MMP-9, CCR2, and CXCR4. See also Supplementary Figure 1.*

Moreover, as shown in Supplementary Figure 1, to better distinguish the novel monocyte population of PCM from the known classification of CM, IM, and NCM, flow cytometry characterization of this subset was performed in patients presenting with NSTE-ACS (*n* = 5), in reliance on the expression of the following surface markers: CD14, CD16, CD45, CD69, MMP-9, CCR2, and CXCR4 (Table 2).

### Distribution of Circulating Monocyte Subsets in Non-ST-Elevation Acute Coronary Syndrome Patients and Its Relationship With Plaque Phenotype (Erosion Versus Rupture) and Local Macrophage Infiltration at Optical Coherence Tomography Investigation

**FIGURE 3:**
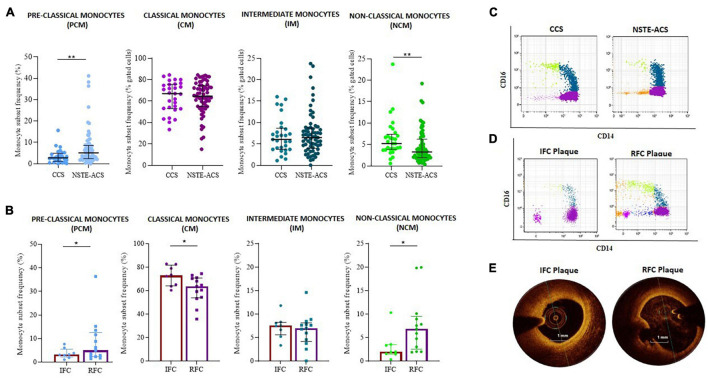
Distribution of monocyte subsets between chronic coronary syndrome (CCS) and non-ST elevation acute coronary syndrome (NSTE-ACS) patients and its relationship with plaque phenotype at optical coherence tomography (OCT) investigation. **(A)** Frequency of pre-classical, classical, intermediate, and non-classical monocytes in CCS (*n* = 30) and NSTE-ACS (*n* = 69) patients and **(B)** in NSTE-ACS patients with evidence of intact fibrous cap (IFC, *n* = 8) and ruptured fibrous cap (RFC, *n* = 13) plaques according to OCT investigation. Two representative dot plots showing monocyte subset distribution **(C)** in CCS and NSTE-ACS patients and **(D)** in NSTE-ACS patients presenting with IFC and RFC plaques. **(E)** Two representative OCT images of IFC and RFC plaques. Data are presented as mean ± SD; *p < 0.05, **p < 0.01.

Interestingly, when monocyte subset distribution was evaluated in the whole study population, the newly identified PCM subset was more frequent in NSTE-ACS rather than in CCS patients (median, IQR: 5.2, 6.2 vs. 2.8, 3.9; *p* = 0.005). NCM was more frequent in CCS rather than in NSTE-ACS patients (median, IQR: 5.2, 3.6 vs. 3.3, 4.3; *p* = 0.006), while no significant differences were recorded comparing the CM (median, IQR: 67.0, 22.7 vs. 64.6, 19.6; *p* = ns) and IM (median, IQR: 6.1, 4.9 vs. 6.5, 4.8; *p* = ns) frequency distributions between CCS and NSTE-ACS patients (*p* > 0.999 for both). Nevertheless, CM was the most frequent monocyte subgroup in both CCS and NSTE-ACS, as expected according to the known monocyte subset distribution (Figure 3A).

When plaque phenotype was analyzed by OCT, NSTE-ACS patients with RFC plaques (*n* = 13) compared to patients with IFC plaque (*n* = 8) displayed a decreased frequency of CM (mean ± SD: 60.1 ± 13.4 vs. 74.5 ± 7.8, *p* = 0.013) and an increased frequency of NCM (median, IQR: 6.9, 7 vs. 2.0 2.0, *p* = 0.008). IM frequencies were similar between erosion and ruptured plaques (*p* = 0.886). The same analysis showed an increasing frequency of PCM in NSTE-ACS patients presenting with RFC as compared with IFC lesions (median, IQR: 5.0, 10.1 vs. 3.2, 3.4; *p* = 0.054) (Figure 3B). Representative dot plots of monocyte subset distribution in CCS and NSTE-ACS patients (Figure 3C) and in NSTE-ACS according to plaque phenotype (Figure 3D), and representative OCT images (Figure 3E) are shown.

**FIGURE 4:**
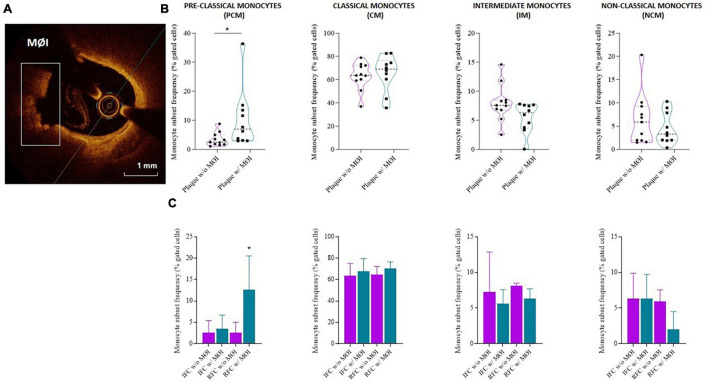
Distribution of monocyte subsets in non-ST elevation acute coronary syndrome (NSTE-ACS) patients according to plaque erosion (IFC) or rupture (RFC) and local macrophage infiltration (MØI). **(A)** Representative optical coherence tomography (OCT) image of MØI. **(B)** Frequency of circulating monocyte subsets in NSTE-ACS patients according to OCT-MØI assessment, **(C)** and according to both plaque morphology (IFC *versus* RFC) and MØI. Data are presented for panel **(B)** as median and interquartile range and for panel **(C)** as mean ± SD; *p < 0.05.

Higher frequency of PCM was recorded in NSTE-ACS patients presenting plaque MØI as compared to patients without local MØI (median, IQR: 7, 10.1 vs. 2.5, 3.3 *p* = 0.013), and specifically in NSTE-ACS patients with RFC lesions and MØI as compared to those with RFC lesions without MØI (median, IQR: 12.6, 15.1 vs. 2.5, 3.3; *p* = 0.011), while no significant differences were recorded comparing the other three monocyte subsets (Figures 4A–C).

### In Non-ST-Elevation Acute Coronary Syndrome Patients With Plaque Rupture, Lipopolysaccharide-Stimulation of Peripheral Blood Mononuclear Cells Triggers an Enhanced Cytokine Release

**FIGURE 5:**
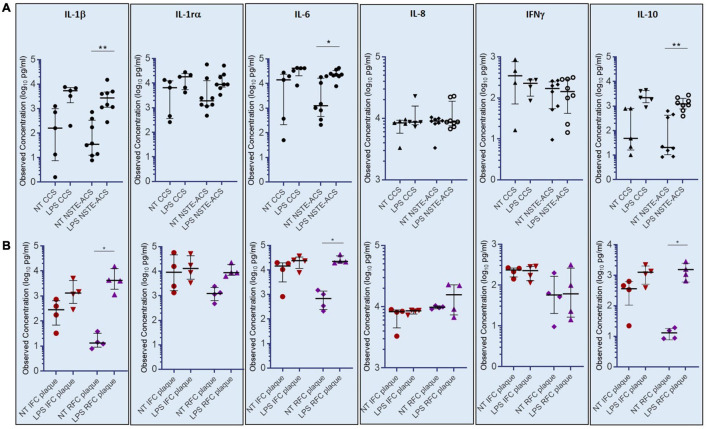
Levels of cytokines (IL-1β, IL-6, and IL-10) assessed in supernatants of cultured peripheral blood mononuclear cells (PBMCs) not-treated or treated with *E. coli* lipopolysaccharide (LPS). **(A)** Histograms showing observed cytokine concentrations (pg/ml) in the supernatant of chronic coronary syndrome (CCS) and non-ST-elevation acute coronary syndrome (NSTE-ACS) PBMCs. **(B)** Histograms showing observed cytokine concentrations (pg/ml) in NSTE-ACS patients underwent to optical coherence tomography (OCT) investigation. Data are presented as median and interquartile range; *p < 0.05, **p < 0.01.

The assessment of a cytokine panel by multiplex ELISA revealed that cultured PBMCs from NSTE-ACS patients respond to LPS treatment by releasing significant amounts of IL-1β, IL-6, and IL-10 in the cell culture medium, as compared with non-treated cells (NT) (*p* = 0.008; *p* = 0.016; *p* = 0.008, respectively), while no differences have been recorded for IL-1rα, IL-8, and INF-γ (Figure 5A). Although showing a release of IL-1β, IL-6, and IL-10 in response to LPS stimulation, no significant differences has been recorded between NT and LPS-treated PBMCs from CCS patients for all the six cytokines studied. According to OCT analysis, the magnitude of LPS-mediated effects on IL1-β, IL-6, and IL-10 release in cell culture medium appeared to be greater in patients with RFC (*n* = 6), when compared to patients with IFC (*n* = 4), hence showing that these data have plaque specificity. Indeed, supernatants from PBMCs of RFC patients released the highest levels of IL-1β (*p* = 0.035), IL-6 (*p* = 0.012), and IL-10 (*p* = 0.014) after LPS treatment, while no significant differences have been recorded within the IFC group (Figure 5B). No significant differences were found in high-sensitivity C-reactive protein (hs-CRP) levels comparing IFC and RFC patient samples (see Supplementary Figure 2).

## Discussion

The pathophysiology of ACS is strictly related to an outburst of inflammatory (Biasucci et al., 2017) and immune-mediated response (Crea and Liuzzo, 2013), taking over the concept of the atheroma as a mere lipid-driven lesion (Soehnlein and Libby, 2021). Differences in innate and adaptive immune system have been described in ACS patients (Liuzzo et al., 2007, 2020; Flego et al., 2016; Leers et al., 2017; Pedicino et al., 2018a; Leistner et al., 2020). Starting from PBMCs as source material, our group documented an altered hyaluronan metabolism in NSTE-ACS patients presenting with plaque erosion (Pedicino et al., 2018b), further confirmed by later investigations on platelets and monocyte-platelet interaction (Vinci et al., 2021). Those findings pointed the attention to the extracellular matrix derangement and its altered components (Angelini et al., 2018). On the other side, an expansion of CD4^+^CD28^null^ T-cells represents distinctive features of NSTE-ACS patients with RFC lesions (Ruggio et al., 2019). These data suggest peculiar pro-inflammatory and immune-mediated mechanisms underneath different plaque morphologies.

Monocytes, as immune system early players, can receive alerts by infectious antigens through their surface pattern recognition receptors (PRRs) (Zanoni and Granucci, 2013) as well as by systemic and local pro-inflammatory signals (Shi and Pamer, 2011). Circulating monocyte subsets, whose detection is based on their surface expression of CD14 and CD16 antigens (Ziegler-Heitbrock et al., 2010), might have a variable distribution depending on the pathophysiological condition and their distinctive ability to plastic self-conversion (Gerrity, 1981; Stec et al., 2007; Kratofil et al., 2017; Roberts et al., 2020). Maladaptive innate immune plasticity, already recognized as a pivotal player in autoimmune diseases such as rheumatoid arthritis (Xue et al., 2020), systemic sclerosis, and systemic lupus erythematosus syndromes (Hirose et al., 2019; Ma et al., 2019), might fuel plaque fate and acute coronary events.

According to our multicolor flow-cytometry analyses, the decreased frequency of NCM in the peripheral blood of our NSTE-ACS patients as compared to CCS ones, beforehand proved by Flego et al. (2015), corroborated the downregulation of patrolling monocytes during an acute coronary event (Thomas et al., 2015; Leers et al., 2017). With this study, we aimed at investigating monocyte population distribution according to atherosclerotic plaque phenotyping.

Our data showed that NSTE-ACS patients presenting with RFC plaques exhibit a fall in the frequency of CM subset, alongside higher levels of circulating NCM. These findings might reflect a compensatory mechanism of CM consumption, on one side, and an increased demand of circulating NCM, on the other side, that, all in all, could serve as injury responders (Marsh et al., 2017). Our data are in line with the recent observation of the prevalence of pro-inflammatory monocyte-derived macrophages in ACS patients with plaque rupture (Fracassi et al., 2021).

Intriguingly, for the first time, we described a novel monocyte population in proximity of CM that we named *pre-classical* monocyte. Our novel population resembles a recently identified monocyte subset defined as small CD14^+^ CD16^neg^ through a comprehensive multicolor flow-cytometry study on healthy subjects (Merah-Mourah et al., 2020). The newly described PCM subset was higher in NSTE-ACS patients, in particular with RFC plaque and with concomitant presence of local macrophage infiltration (MØI) as assessed by OCT investigation. MØI represents one of the main initiators of atherosclerotic plaque onset and progression. Our group has previously demonstrated that not all ACS patients with plaque rupture have local MØI, and that MØI is closely related with systemic inflammation (Scalone et al., 2017). Here we added another missing piece in the puzzle. Indeed, we found no differences in systemic hs-CRP levels between IFC and RFC, suggesting a similar systemic inflammatory burden in the two groups of patients. However, PCM expansion is strictly related to both MØI and RFC, while similar frequencies were observed in IFC and CCS, indicating an important role for this monocyte subset in the pathogenesis of plaque rupture at least in part independent from the systemic inflammatory burden (Biasucci et al., 2020). These data strongly suggest that alongside well recognized triggers of instability, such as systemic inflammation, other players are involved in the different fate of unstable plaque and that PCM might be used both as signature of plaque rupture and as promising targets for future therapeutic tools.

Finally, the LPS-treatment of circulating PBMCs of NSTE-ACS patients affects the pro-inflammatory (IL-1β, IL-6) as well as anti-inflammatory (IL-10) cytokine release in cell-culture supernatants, especially in patients with RFC, suggesting that the intensity of the individual response to potential inflammatory stimuli may play a major role in determining the magnitude of the inflammatory reaction and clinical outcome (Liuzzo et al., 2001). These *in vitro* findings support our previous observation that NSTE-ACS with RFC are characterized by the expansion of an unusual subset of T cells committed to production of immunomodulatory cytokines, such as IL-12 and interferon (IFN)-γ (Ruggio et al., 2019). As these cytokines induce a rapid priming of human monocyte functions, their chronic up-regulation could lead to subsequent activation of monocytes/macrophages in the circulation as well as in tissue lesions.

## Study Limitations

Our study has some limitations. First, a formal estimate of the sample size was not pursued due to the innovative nature of the study. Since our work is a prospective analysis that includes a limited number of patients, we cannot exclude that the biological alterations described above may be part of the general stress response in the acute phase of ACS. Second, we based our plaque characterization on a small subset of enrolled NSTE-ACS patients who underwent OCT estimation for clinical reasons; study inclusion was also limited by the diagnostic certainty of OCT readers. Third, we tested the difference in inflammatory response following an exogenous pro-inflammatory stimulus, such as LPS, in patients with eroded and ruptured plaque, although we were aware of the small number of patients involved in this *in vitro* experiment due to sample availability and its allocation within each experimental phase. Finally, as the detection of macrophages by OCT raises technical limitations and consequent misdiagnoses, further validations with autopsy studies could be useful to confirm these results. We are aware that this is a descriptive and rather limited work, which needs to be confirmed in larger studies, and that the pathophysiology of our novel monocyte subset still needs to be properly investigated, conceivably using the single-cell technologies.

## Conclusion

Circulating monocyte subsets of patients with NSTE-ACS show phenotypic heterogeneity with downregulation of patrolling monocytes and prevalence of inflammatory features in case of plaque rupture. Thus, monocyte subset diversity may take part in the cellular pathways leading to fibrous cap rupture and subsequent thrombus formation.

Of note, the present study is the first to investigate the pathophysiological role of a novel monocyte subpopulation that we named *pre-classical* monocyte (PCM) and to highlight a close relationship between PCM and plaque rupture with local macrophage infiltration. Alongside, the similar level of systemic inflammatory burden between patients presenting with RFC and IFC plaques, as indicated by CRP levels, confirms that mechanisms underlying plaque instability might be multiple and sometimes scarcely dependent on inflammatory bursts. In the era of precision medicine, different pathogenetic pathways might lead to ACS, and in this scenario, the higher frequency of PCM in patients with plaque rupture and local macrophage infiltration might represent a distinctive marker of a specific plaque phenotype, paving the way to novel diagnostic tools and tailored therapies for a selected group of patients. Indeed, in a translational perspective, the availability of a rapid blood test as a parallel biological, non-invasive screening that estimates the frequency of PCM during the patient hospital admission might be helpful for the consequent allocation of each patient toward even more personalized pharmacological therapies and/or interventional approaches (Crea and Liuzzo, 2016). Further specific studies are warranted in order to support our hypothesis.

## Data Availability Statement

The original contributions presented in the study are included in the article/Supplementary Material, further inquiries can be directed to the corresponding author/s.

## Ethics Statement

The studies involving human participants were reviewed and approved by Ethics Committee of the Fondazione Policlinico Universitario “Agostino Gemelli” IRCCS– Università Cattolica del Sacro Cuore (UCSC), Rome (IT). The patients/participants provided their written informed consent to participate in this study.

## Author Contributions

RV and DP: conception and design. PC, MDS, CC, and FCr: provision of study materials and enrollment of patients. RV, AB, EP, AS, and FCa: collection of biological data and experimental activities. RV, DP, and GL: assembly of biological data. AD’A, MP, and MCG: collection and assembly of clinical data. CT, GR, and RM: hemodynamics investigation. RV, DP, and GL: data analysis and interpretation. GL, MM, and FiC: supervision. All authors approved the manuscript.

## Conflict of Interest

The authors declare that the research was conducted in the absence of any commercial or financial relationships that could be construed as a potential conflict of interest.

## Publisher’s Note

All claims expressed in this article are solely those of the authors and do not necessarily represent those of their affiliated organizations, or those of the publisher, the editors and the reviewers. Any product that may be evaluated in this article, or claim that may be made by its manufacturer, is not guaranteed or endorsed by the publisher.
