# Supplementary material for: A Novel Monocyte Subset as a Unique Signature of Atherosclerotic Plaque Rupture
Source: Front Cell Dev Biol. 2021 Oct 12;9:753223. doi: 10.3389/fcell.2021.753223 (PMC8545820; doi:10.3389/fcell.2021.753223)
Supplement: Supplementary file 1 [file Data_Sheet_1.docx]

Supplementary Material

# Supplementary Figures


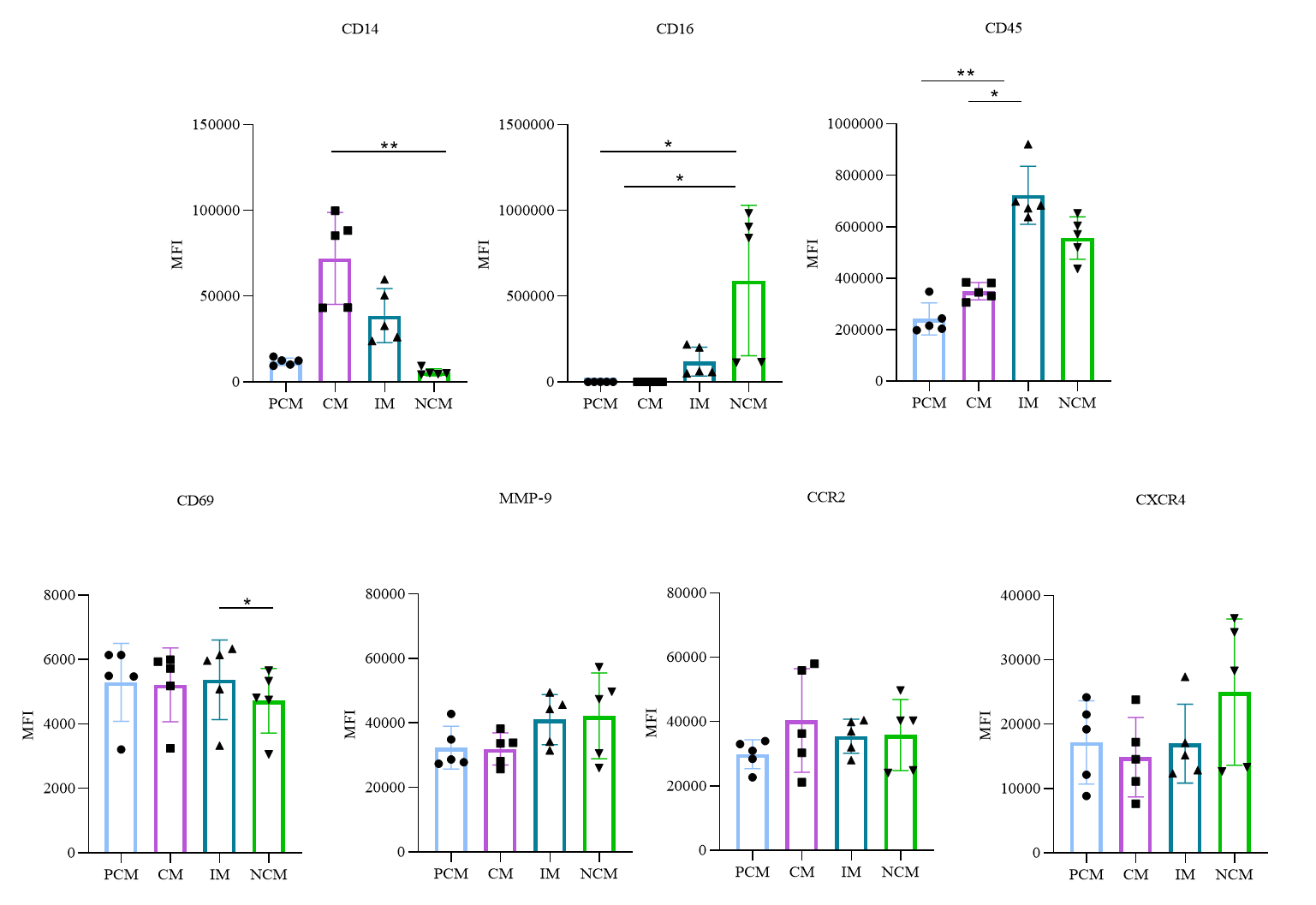


**Supplementary Figure 1.** Flow cytometry characterization of PCM subset.

Flow-cytometry characterization of monocyte subsets was performed in five patients presenting with ACS, according to the expression of the following surface markers: CD14, CD16, CD45, CD69, MMP-9, CCR2, and CXCR4. CD14 was highly expressed in CM as compared with the other three subsets; a significant difference in CD14 expression was observed between CM and NCM (p = 0.001); CD14 expression was also higher in CM than in PCM (p = 0.086). CD16 was highly expressed in NCM, and absent in PCM and CM. CD45 was significantly higher in IM as compared with CM and PCM (IM *vs* CM, p = 0.042; IM *vs* PCM, p = 0.004). Although expressed in all subsets, CD69 was significantly higher in IM as compared with NCM (p = 0.042). For the other surface markers (MMP-9, CCR2, and CXCR4) we did not record any differences.


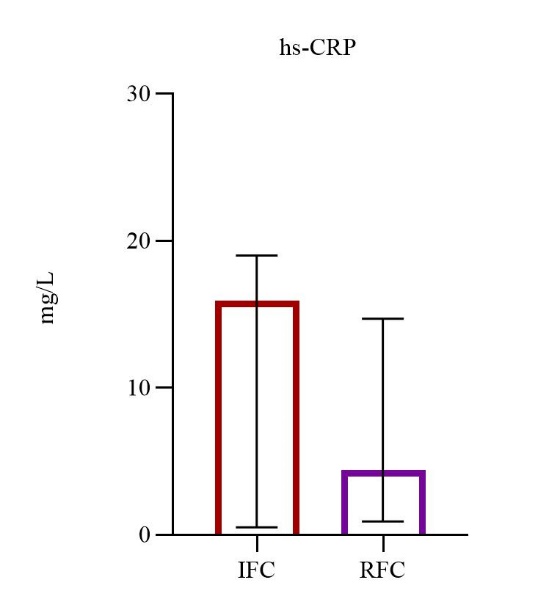
**Supplementary Figure 2.** High sensitive C-reactive protein (hs-CRP) plasma levels dichotomized according OCT-phenotype in NSTE-ACS patients. No statistically significant differences were recorded between plaque erosion (IFC) and rupture (RFC). IFC median value = 15,9 (IQR: 18.5), RFC median value 4,4 (IQR: 13,8); p = 0.705. Data are expressed in median values and interquartile ranges (IQR).
